# Fetal Bowel Dilatation: A Sonographic Sign of Uncertain Prognosis

**DOI:** 10.1155/2015/608787

**Published:** 2015-12-24

**Authors:** Patrícia Silva, Filipa Reis, Paulo Alves, Luís Farinha, Manuel Sousa Gomes, Pilar Câmara

**Affiliations:** ^1^Obstetrics and Gynecology Department, Dr. Nélio Mendonça Hospital, Luís de Camões, Funchal, 9004-514 Madeira Island, Portugal; ^2^Pediatric Surgery Department, Dr. Nélio Mendonça Hospital, Luís de Camões, Funchal, 9004-514 Madeira Island, Portugal

## Abstract

Fetal bowel dilatation is an indirect sonographic sign of mechanical or functional bowel obstruction. The etiology of fetal bowel dilatation is a difficult prenatal diagnosis since ultrasound has limited accuracy for bowel evaluation. The authors describe a case of fetal bowel dilatation diagnosed in the third trimester.

## 1. Introduction

Fetal bowel dilatation is characterized by fluid-filled intestinal loops which measure at least 15 mm in length or 7 mm in diameter [1]. Ultrasonographic image of dilated fetal bowel is a sign of intestinal mechanical or functional obstruction and its prevalence will depend on the underlying condition: bowel atresia or stenosis, malrotation with volvulus, meconium ileus, total colonic aganglionosis, and meconium plug syndrome [2].

The diagnosis of an intestinal dilatation cause is difficult. Some obstructions may not be seen until the late second trimester [3]. The difference between dilated small bowel loops and colon by ultrasound imaging is challenging as is the accurate identification of the number and location of obstructions [4, 5].

As such, fetal bowel dilatation might be associated with different postnatal outcomes which makes the prenatal management and parental counseling problematic.

The authors describe a case of fetal bowel dilatation in the third trimester, followed by a brief review of differential diagnosis and management.

## 2. Presentation of the Case

**Figure 1:**
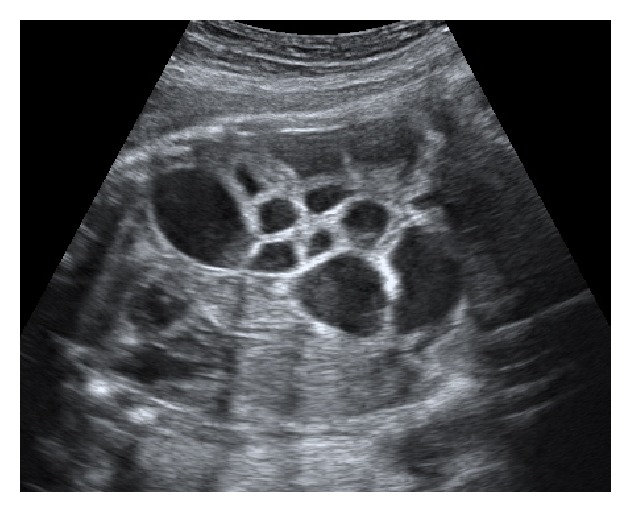
Dilated bowel loops seen in 30th-week gestation.

**Figure 2:**
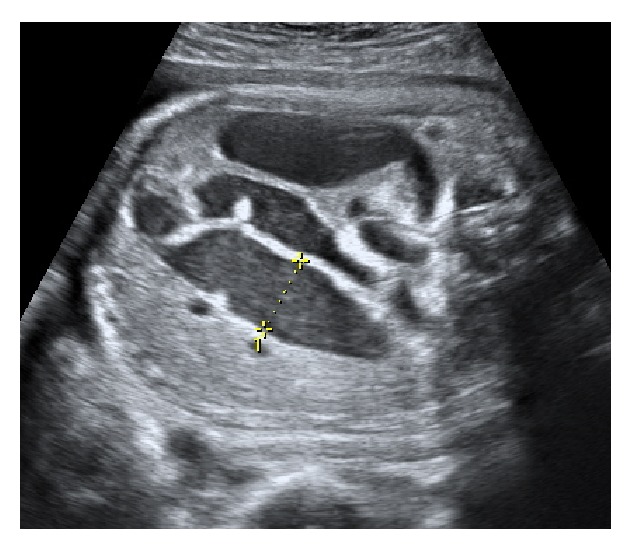
Intestinal loop dilatation with 18 mm.

A 32-year-old healthy primigravida with a normal pregnancy was scheduled for her 30th-week obstetric evaluation. The ultrasound showed a single fetus in breech presentation, with a normal weight for gestational age and with lower gastrointestinal (GI) tract dilatation (Figures 1 and 2). Peristaltic movements were present, the amniotic fluid index was normal, and no other fetal anatomic anomalies were observed.

**Figure 3:**
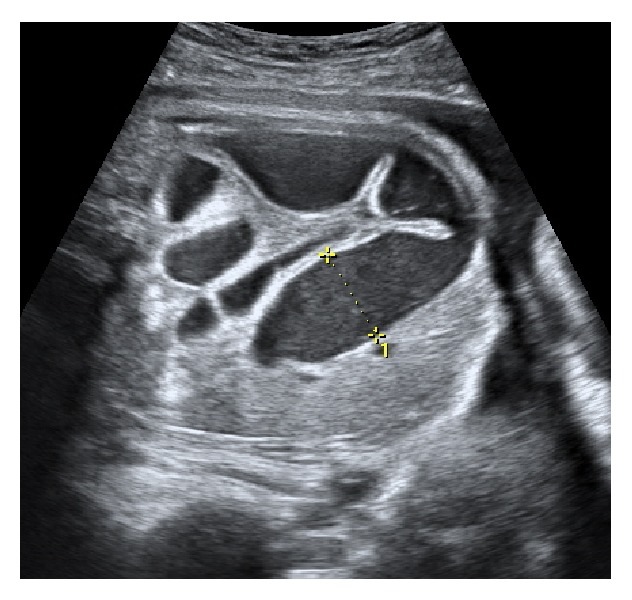
Intestinal loop dilatation with 23 mm at 33rd week.

This anomaly was monitored weekly, showing a progressive increase in size (Figure 3). The amniotic fluid was still normal and at the 33rd week the patient underwent fetal lung maturation.

**Figure 4:**
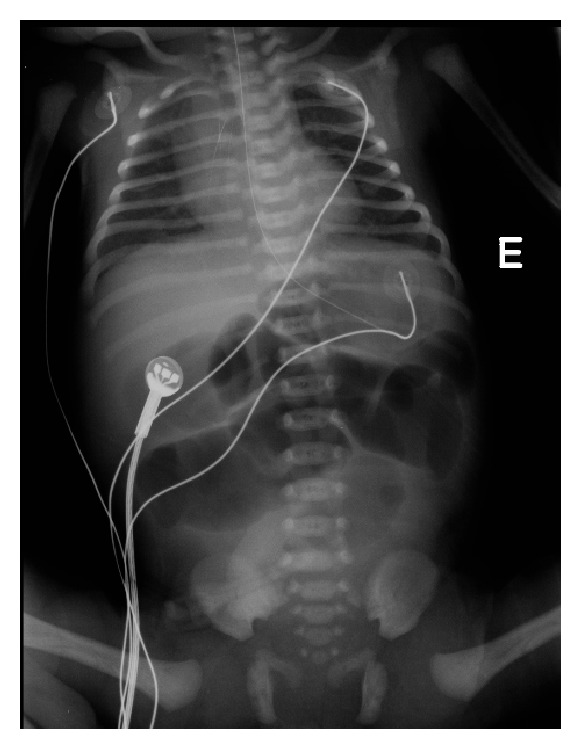
Intestinal distension and air fluid levels.

The case was discussed with the Pediatric Surgery Department and an elective cesarian was scheduled at the 36th week. However, at the 35th week, the patient had spontaneous membrane rupture and so a cesarian was promptly made. The result was a female newborn weighing 2400 gr with Apgar score of 9 on the first minute and 10 on the fifth. The physical examination showed no abdominal distension, masses, visceromegaly, or other malformations. The anal orifice, pharynx, and lower oesophagus were not obstructed. Abdominal X-ray showed distended bowel and air fluid levels with possible extension until the jejunum (Figure 4), probably indicating lower intestinal atresia.

**Figure 5:**
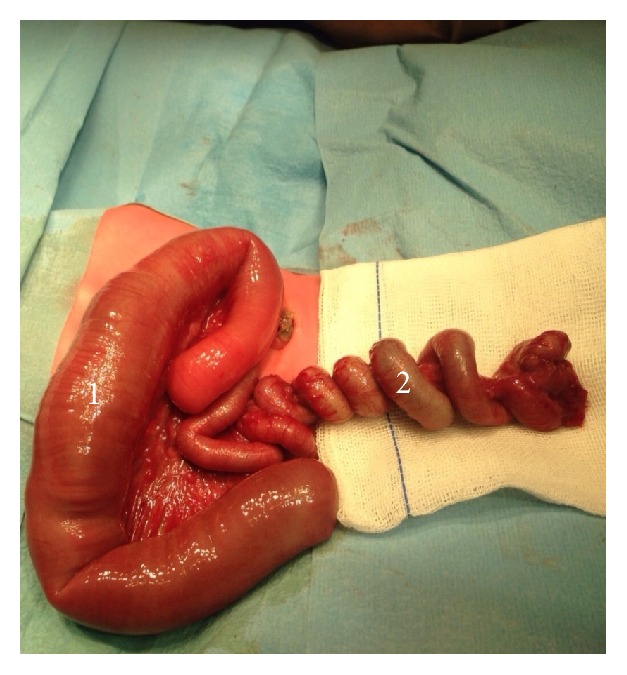
1, proximal dilated ileum loop ending in a blind pouch. 2, atresic “apple peel” distal portion with approximately 45 cm.

**Figure 6:**
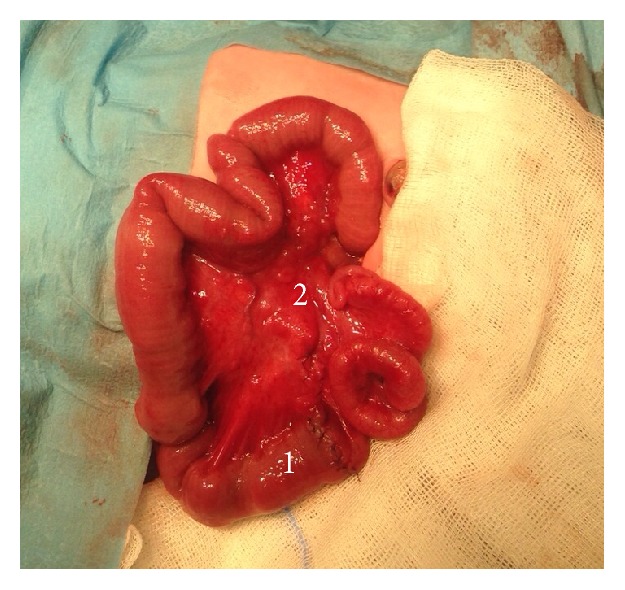
1, end-to-end jejunoileal anastomosis. 2, ileocecal valve.

Exploratory laparotomy was performed on the second day of life and a proximal dilated ileum loop followed by an atresic “apple peel” portion, with approximately 45 cm, which terminated in 30 cm normal ileum was seen (Figure 5). Small bowel resection with end-to-end jejunoileal anastomosis was performed. 80 cm of normal ileum as well as the ileocecal valve was left in place (Figure 6).

**Figure 7:**
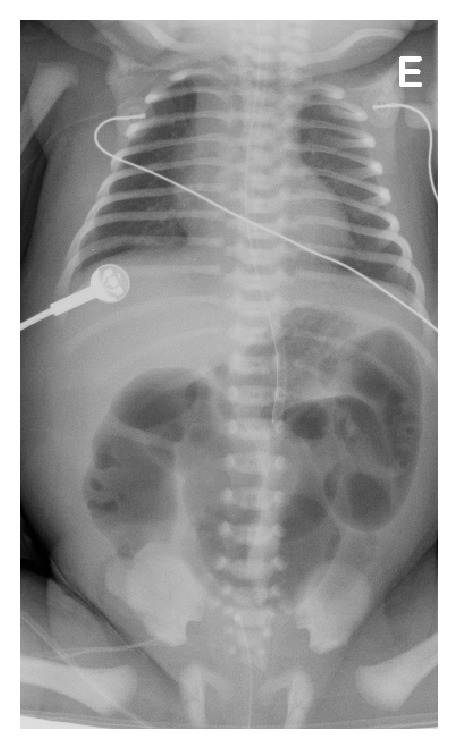
Intestinal distension with air fluid levels and stop sign at the jejunoileal transition.

During the postoperative period, the newborn was apyretic and eupneic and remained on parenteral nutrition. On the 5th day, abdominal X-ray revealed intestinal dilatation with an obstruction at the level of the jejunoileal transition (Figure 7). New laparotomy showed omental adhesion near the jejunoileal anastomosis, which was functional. Given the need to start oral feeding and the likelihood of a new occlusion, ileostomy and adhesiolysis were performed.

After surgery, the newborn was clinically stable, with no changes in the intestinal transit and with progressive weight gain. An end-to-end jejunoileal anastomosis was carried out eighteen days later.

At the 30th day of life, the patient was discharged from the intensive care unit and is currently 2 months old and asymptomatic and with a normal height and growth for her age.

## 3. Discussion

The authors report a case of “apple peel” ileal atresia diagnosed after postnatal investigation of fetal intestinal dilatation. Bowel atresia is a common surgical cause of neonatal GI obstruction [6]. Jejunoileum is the most affected intestinal segment, with incidences ranging from 1 in 1500 to 12000 births. Duodenal atresia occurs in one in 10000 to 40000 followed by the colon, which is the least affected portion, with an incidence of approximately 1 in 40000 live births [7, 8].

Jejunal and ileal atresia (JIA) have been classified into four types based upon their anatomic characteristics [4]. Type IIIB, also known as apple peel, is a rare form and accounts for 11% of the JIA [7]. It is considered to be a consequence of interruption of mesenteric blood flow in early stages of gestation, which in turn causes an embryonic anomaly of the affected segment [9]. The intestinal portion distal to the malformation is foreshortened and coiled like an apple peel [7]. The maternal use of vasoconstrictive medications and drugs, inherited thrombophilias, and fetal malformations which lead to vascular blood disruption (like gastroschisis) could be implicated in its pathogenesis [10–12]. Despite being predominantly sporadic, hereditary cases of JIA have been reported, suggesting a genetic etiology [13].

Prenatal ultrasound findings of intestinal obstruction consist of visualization of dilated bowel loops on ultrasound [9], and this finding was reported at the 30th week in the presented case.

The ability to diagnose GI atresia prenatally is influenced by the gestational age, the site of obstruction, and the presence of associated anomalies.

Concerning the first two, before 24 weeks' gestation, bowel loops are not recognized because there is no efficient gastric peristalsis [2]. After week 25, the bowel becomes echogenic, similar in echogenicity to adjacent liver [3]. Fetal swallowing with passive and active gastric emptying produces the fluid filling of small bowel loops and the accumulation of meconium throughout the second and third trimesters [2]. In this case and like others described in the literature [9], the bowel dilatation only became evident in the third trimester. Concerning the intestinal segment affected, ultrasonographic distinction between small and large bowel is troublesome; however, there are some signs that may lead to a more proximal or distal obstruction [14]. The “double bubble” sign which includes a dilated fluid-filled stomach adjacent to a dilated proximal intestinal segment indicates a contiguous obstruction, such as duodenal atresia [15]. The amniotic fluid volume is higher in the most proximal obstructions. The presence of an enlarged stomach and polyhydramnios is consistent with jejunal rather than ileal atresia [16]. Finally, when the colon is obstructed, there is no bowel dilatation generally, because fluid is resorbed in the upstream small bowel and colonic loops [4].

The prenatal management of suspected small bowel distal atresia includes the fetal evaluation for associated disorders and unlike duodenal atresia and colonic obstructive disorders, aneuploidy and extraintestinal malformations are unusual [4]. However, GI anomalies are present in up to 45% of cases and may occur simultaneously, such as esophageal atresia (3%), or may play a role in the etiology of the obstruction, such as malrotation (23%), meconium ileus (10%), or gastroschisis [4]. Though some evidence points to a benefit in using magnetic resonance as a complement in the assessment of bowel obstruction, it is not consensual [7] and in this clinical setting it would not change the obstetrical conduct.

In the reported case, there is isolated fetal bowel dilatation in an otherwise anatomically normal growing fetus, so the need for other diagnostic evaluation is secondary once it does not add further value to management or care of the mother or fetus.

Follow-up ultrasound examinations were performed. Bowel appearance and associated complications, like perforation, ascites, meconium peritonitis, or meconium pseudocysts, were weekly monitored as well as fetal growth and amniotic fluid volume. The postnatal investigation confirmed a distal small bowel obstruction due to “apple peel” ileal atresia.

Despite low rate of sensibility and specificity, ultrasound plays an important role in the management and diagnosis of fetal bowel dilatation. It offers an opportunity for parental counseling and for choosing patients who need transfer to a specialized center which is of upmost importance as it allows prompt treatment and reduces the risk of complications.
